# Genetic testing and evidence of a founder mutation in a hotspot for hereditary transthyretin amyloidosis

**DOI:** 10.1038/s41598-025-14707-4

**Published:** 2025-08-14

**Authors:** Marc Ventayol-Guirado, Eugenia Cisneros-Barroso, Maria Antonia Ribot-Sanso, Juan Gonzalez-Moreno, Ines Losada, Tomas Ripoll-Vera, Jaume Pons, Elena Fortuny, Teresa Bosch, Antonio Figuerola, Cristina Descals, Joan Carles Montala, Jorge Alvarez-Rubio, Jessica Hernandez-Rodriguez, Jose Lustre-Rodriguez, Maria Victoria Llull-Alberti, Juan Antonio Jimenez-Barcelo, Victor Jose Asensio-Landa, Laura Torres-Juan, Iciar Martinez-Lopez, Juan Buades-Reines, Damian Heine-Suñer

**Affiliations:** 1 Genomics of Health Research Group, Health Research Institute of the Balearic Islands (IdISBa), Palma, Balearic Islands Spain; 2 Balearic Research Group in Genetic Cardiopathies, Sudden Death and TTR Amyloidosis. Health Research Institute of the Balearic Islands (IdISBa), Palma, Balearic Islands Spain; 3 Internal Medicine Department, Son Llàtzer University Hospital, Palma, Balearic Islands Spain; 4 Cardiology Department, Son Llàtzer University Hospital, Palma, Balearic Islands Spain; 5 Cardiology Department, Son Espases University Hospital, Palma, Balearic Islands Spain; 6 Internal Medicine Department, Son Espases University Hospital, Palma, Balearic Islands Spain; 7 Neurology Department, Son Llàtzer University Hospital, Palma, Balearic Islands Spain; 8 Molecular Diagnostics and Clinical Genetics Unit, Son Espases University Hospital, Palma, Balearic Islands Spain; 9 Genosalut, Palmaplanas Quiron, Palma, Balearic Islands Spain

**Keywords:** *TTR*, Variant transthyretin (ATTRv) amyloidosis, Genetic testing, Founder event, V30M, Referral services, Clinical genetics, Medical genetics, Cardiomyopathies, Peripheral neuropathies

## Abstract

Variant transthyretin (ATTRv) amyloidosis is a rare, inherited disorder caused by mutations in the *TTR* gene, leading to amyloid fibril deposition. The Balearic Islands are a known endemic focus for the NP_000362.1:p.Val50Met (V30M) variant, one of the most prevalent pathogenic mutations. We analysed 23 years of genetic testing data from the Balearic Islands’ public health system to determine the prevalence and distribution of ATTR pathogenic variants, with a focus on V30M. A total of 1,478 individuals underwent genetic testing, with 319 positive tests for ATTRv: 308 carried the V30M variant (96.4%) and 11 carried other pathogenic variants. The V30M prevalence was highest in Mallorca and Menorca (1 in 2,900 and 4,700, respectively), with lower rates in Ibiza and Formentera. The co-occurrence of V30M and NP_000362.1:p.Gly26Ser (G6S) on the same chromosome in 93% of V30M carriers suggests a common origin. This study becomes the first registry of ATTRv in the Balearic Islands, aiming to raise awareness among clinicians across all areas and services while enabling more accurate diagnostics, informed genetic counselling, and targeted clinical follow-ups. We also postulate the Balearic Islands as a major global focus for the V30M variant, with a distinct genetic profile suggesting a unique founder effect within the region.

**Supplementary Information:**

The online version contains supplementary material available at 10.1038/s41598-025-14707-4.

## Introduction

Variant transthyretin (ATTRv) amyloidosis is a rare and life-threatening disease with roots tracing back several centuries. Although its clinical manifestations were observed as early as the 17th century, it was not until 1952 that Corino Andrade provided key insights into the pathogenesis and epidemiology of ATTRv amyloidosis^1–3^. Subsequent research identified amyloidogenic transthyretin (ATTR), originally termed prealbumin, as the primary amyloid protein responsible of the disease^4^and pinpointed the causative NP_000362.1:p.Val50Met (V30M) mutation^5–7^ on chromosome 18 ^8,9^. This mutation was found to occur independently in different populations, despite hypotheses of historical transmission^10^. Until 1990, V30M ATTR amyloidosis was considered an endemic disease primarily affecting northern Portugal, northern Sweden, and two regions in Japan, with a prevalence of 1–10 per 10,000 inhabitants^11–13^. However, since then, other endemic foci for the V30M ATTR amyloidosis have been identified, including Mallorca (Balearic Islands)^14^.

The epidemiology of ATTRv amyloidosis in Spain, particularly in the Balearic Islands, presents a complex and evolving landscape. Nationwide prevalence data are scarce, with current estimates likely underrepresenting the true burden of disease due to the absence of comprehensive national registries. Despite this, certain endemic foci such as Mallorca are well-documented. In 1988, Munar-Qués identified 47 Mallorcan patients with the V30M variant and what was then known as familial amyloid polyneuropathy type I^14^. Subsequent studies from the Multidisciplinary ATTRv Unit at Son Llàtzer University Hospital have further characterized this population, revealing significant insights into disease demographics and clinical presentation^15–17^. In 2016, a study of 95 patients with V30M ATTR amyloidosis found that 55.7% had early-onset disease, while 44.3% had late-onset^16^. By 2020, a broader study had identified 79 living patients and 108 living asymptomatic V30M carriers, suggesting a higher prevalence than previously reported^17^. These studies highlight a predominance of early-onset forms with an average onset age of 47 years. Additionally, new Spanish endemic foci have been identified, such as in Huelva^18^ with the V30M variant and in Jaen with the NP_000362.1:p.Glu109Lys (E89K) variant^19^with the latter also being associated with a mixed phenotype. The V30M variant remains the most frequent variant in Spain, reflecting its prevalence in the above-mentioned endemic areas.

V30M ATTR amyloidosis can affect multiple organs and systems, including the nervous and gastrointestinal systems, the heart, kidneys and eyes. It presents in many different forms and with considerable variation in signs and symptoms across individuals and geographic locations^20^. The clinical features of V30M ATTR amyloidosis are mainly neuropathic, with a heterogeneous presentation of peripheral (sensory and motor) and autonomic neuropathy. Gastrointestinal impairment, cardiomyopathy, ocular manifestations and nephropathy are other frequent manifestations of the disease^21^.

Over the past two decades, the understanding, diagnosis, and treatment of ATTR amyloidosis have advanced significantly. The once geographically limited disease is now recognized in 29 countries^22^ with over 140 pathogenic variants identified in the *TTR* gene (ENSG00000118271)^23^. In addition, the growing prevalence of wild-type ATTR (ATTRwt) amyloidosis, often diagnosed non-invasively, has spurred increased interest from both researchers and the pharmaceutical industry^24^.

Diagnosis in the early stages of the disease is crucial for timely treatment to prevent progression. However, early recognition remains a challenge, with a significant delay in diagnosis still occurring often due to misdiagnosis^25–30^ especially among those without a family history of the disease. This could be explained in part by the age-dependent penetrance of the gene, the infrequent occurrence of *de novo* mutations or the misdiagnosis of parents^25,26,29–31^. Genetic counselling poses another important challenge for the management of V30M ATTR amyloidosis. While no standard guidelines exist for comprehensive genetic counselling, presymptomatic testing (PST) of the *TTR* gene in family members and, the clinical management of asymptomatic ATTRv carriers identified through an index patient are recommended^32^. This is based on growing evidence highlighting the benefits of early detection and diagnosis in asymptomatic carriers^33,34^.

We present here a review of 23 years of symptomatic and presymptomatic genetic testing for ATTRv amyloidosis in the Balearic Islands.

## Methods

### Study design and population

This study is a retrospective analysis of genetic testing data collected over a 23-year period (2001–2023) within the Balearic Islands public health system. The study population comprised individuals referred for genetic testing either due to clinical suspicion of ATTRv amyloidosis or as part of a PST program based on a positive family history of ATTRv. The PST program is aligned with international recommendations for genetic counselling in this disease^33^ and testing is coordinated by a multidisciplinary team that includes specialists in internal medicine, cardiology, neurology and genetics. Genetic testing is offered to at-risk family members following confirmation of an index case. The process begins with an initial consultation, during which the attending physician provides genetic counselling, documents the family history, explains the nature and implications of ATTRv amyloidosis, and discusses the potential outcomes and consequences of genetic testing. Written informed consent is obtained prior to testing. Results are delivered in person by the attending physician, and for those who test positive, clinical monitoring is established at six- or twelve-month intervals by the multidisciplinary team, including referrals to relevant specialists to facilitate early detection of disease onset and optimize patient outcomes.

This study was performed in line with the principles of the Declaration of Helsinki. Ethical approval was granted by the Ethics Committee of the Balearic Islands and the Research Commission of Hospital Universitario Son Llàtzer (decision number: IB 4641/21 PI), and all patients signed informed consent for research purposes.

### Sample collection and DNA extraction

Peripheral blood samples were collected from all individuals referred for *TTR* genetic testing at various certified blood collection centres across the Balearic Islands over a 23-year period. Genomic DNA was extracted from fresh whole blood using standard protocols in use at the time of collection. DNA extraction methods varied slightly over the years due to changes in available technologies and protocols, evolving from manual extraction using standard kits (Promega Wizard) to the use of automated extractors (Qiagen EZ2 Connect MDX), but all followed validated procedures commonly used in clinical diagnostic laboratories. DNA quality and concentration were assessed using spectrophotometric or fluorometric methods (e.g., NanoDrop or Qubit), depending on the time period and laboratory standards. Only samples meeting minimum quality thresholds were used for downstream genetic analyses.

### Genetic analysis

To identify genetic variants within the *TTR* gene, we performed Sanger sequencing on four PCR products, each corresponding to one of the four exons of the gene. Given the high prevalence of the V30M variant in our region, we adopted a targeted approach, starting with PCR amplification and sequencing of exon 2. If no pathogenic variant was detected in exon 2, the analysis was extended to the remaining three exons. Following sequencing and diagnosis, data on all *TTR* variants were systematically recorded for each individual.

### Co-occurrence analysis

Four patients were randomly selected from carriers for both V30M and NP_000362.1:p.Gly6Ser (G6S) to determine whether these recurrent variants were located on the same chromosome (in cis), as suspected. To investigate this, we performed whole genome sequencing using next-generation sequencing with Illumina technology on a Novaseq 6000 sequencer. The sequencing reads, aligned to the reference genome GRCh38 (bam files), were visualized with the Integrative Genomics Viewer tool^35^. This allowed us to observe whether both variants were found on the same reads, indicating *cis* conformation, or if some reads contained only the V30M variant and others only the G6S variant, indicating *trans* conformation.

### Statistical analysis

We performed chi-squared tests for each referral service to examine the association between sex and V30M positivity. To examine the age distribution, a t-test was performed to compare the mean age of the tested population with that of individuals who tested positive for the V30M variant. The t-test assumptions, including normality and homogeneity of variance, were verified before conducting the analysis. Cases were defined as individuals confirmed to carry the V30M variant. The prevalence of the V30M variant in our region was calculated as the ratio of known alive cases to the total population in the region. The population data for the Balearic Islands, including the individual islands of Mallorca, Menorca, Ibiza and Formentera, was sourced from the most recent regional census conducted in 2023 ^36^.

## Results

This retrospective study involves a diverse cohort of 1,478 individuals, which accounts for more than the 0.1% of the Balearic population, numbering currently as 2023 nearly 1.2 million inhabitants^36^.

Our analysis revealed a significant diagnostic yield, with 319 tests returning positive results, which accounts for 21.6% of the individuals. Among the identified carriers, 308 individuals were found to harbor the V30M variant in heterozygosis, while 11 carriers (3.4%) exhibited other potentially pathogenic variants in the *TTR* gene different from V30M (Supplementary Table 1). No homozygous V30M/V30M cases were identified, despite some cases having been reported in other endemic foci^37^. The V30M variant accounted for 96.6% of the positive results, underscoring its predominance among ATTRv carriers in the Balearic Islands.

**Fig. 1:**
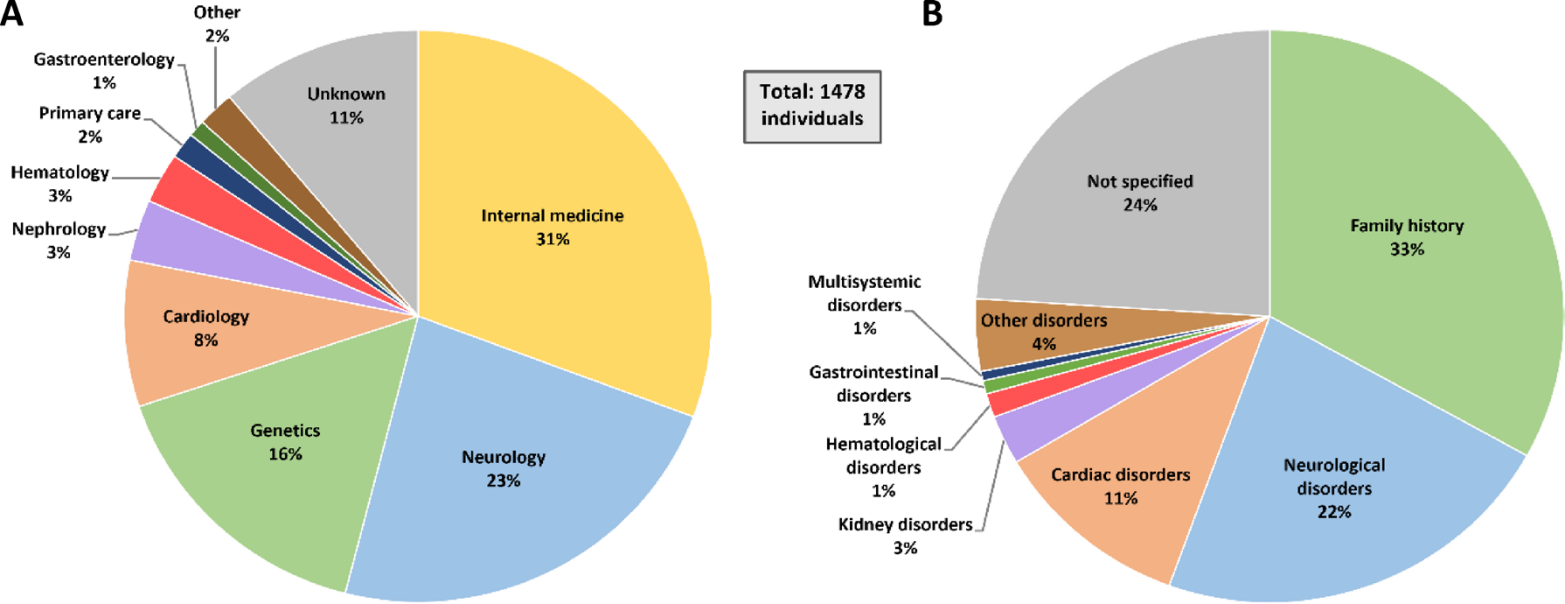
(**A**) Referral services of the tested population. (**B**) Referral reasons of the tested population. Note that the referral service of some individuals was unknown or not specified.

Over the two decades of genetic testing, the detection of ATTRv carriers increased significantly, as illustrated in Supplementary Figure 1. Between 2001 and 2008, fewer than 10 carriers were identified annually. However, from 2012 onwards, there was a notable rise, peaking at over 60 carriers detected in 2019-2020. ATTR amyloidosis patients are often derived for genetic testing from a variety of different referral services. In the present study, referral to genetic testing originated mainly from internal medicine accounting for 31%, neurology for 23%, genetics for 16%, and cardiology for 8% (Figure 1A). Primary referral reasons varied, with the most common being PST for affected relatives (33%) followed by cases involving polyneuropathy or cardiac involvement (Figure 1B).

From this point forward, for consistency, all results will refer exclusively to the 96.6% of individuals with the V30M variant. The demographic analysis of our cohort revealed that 58.1% of the patients were male and 41.9% were female (Table 1). However, when we consider only those positive for V30M, there is a similar percentage of men and women (51.6 vs. 48.4%). We found that women are significantly more likely to test positive for the V30M variant than men, with an overall positive testing rate of 25.6% versus 17.4% (p-value = 1.43·10^− 4^, Chi-square test) (Table 1). If we break down the tested population by referral service from which they were derived (Table 1), we observe the greatest gender differences in those derived from cardiology, as 7.8% of men were positive for V30M versus 25.0% of women (p-value = 0.033, Chi-square test). Thus, we can conclude that the overall significant gender differences in positivity come mainly from patients derived from cardiology and putatively for cardiac-related reasons.

**Table 1:** Demographics of the tested population (sex-dependent).

Referral service	Sex	V30M carriers	Tested individuals	% of positive tests
Internal medicine	Male	57	238	23.9%
	Female	63	216	29.2%
Neurology	Male	20	229	8.7%
	Female	12	113	10.6%
Genetics	Male	42	111	37.8%
	Female	48	127	37.8%
Cardiology	Male	7	90	7.8%
	Female	7	28	25.0% *
Total	Male	149 (48.4%)	858 (58.1%)	17.4%
	Female	159 (51.6%)	620 (41.9%)	25.6% ***
Total	Male & Female	308	1478	**20.8%**

* *p-value < 0.05*, ** *p-value < 0.01*, *** *p-value < 0.001*.

The testing age distribution of individuals in our cohort follows a normal distribution, slightly skewed to older ages, spanning from 18 to 97 years old, with an average age of 58.4. To analyze the age of genetic diagnostics and reduce potential biases, we broke down our cohort into two groups: individuals without known family history of ATTRv and individuals with a family history of the disease. This approach highlighted several interesting traits (Table 2): (1) Younger average age of the cohort of tested individuals with a family history of the disease when compared to those without a family history of the disease (44.5 vs. 64.9 years). (2) The same was observed in those who tested positive for the V30M with or without a family history (43.0 vs. 57.8 years of age). (3) A higher positivity rate for the V30M in younger age groups, which gradually decreases with increasing age in the population without a family history, which is not observed in those with a family history, who show similar positivity rates in all age groups. (4) The age distribution differs between all individuals tested and those found to be V30M carriers in the population without a family history. V30M carriers tend to be diagnosed at an earlier age than the total population tested, with an average age at genetic diagnosis of 57.8 years old and 64.9 years old, respectively (p-value = 1.85·10^− 3^, t-test) (Table 2). (5) This contrasts with what was observed in the group with a known family history of ATTRv (Table 2), where no differences in the age at diagnosis were observed between carriers and tested individuals at 44.5 and 43.0 years, respectively (p-value = 0.372, t-test).

**Table 2:** Demographics of the tested population with and without known family history of ATTRv amyloidosis (age-dependent).

	Age interval	V30M carriers	Tested individuals	% of positive tests
With known family history of ATTRv	18–37	67	136	49.3%
	38–57	61	163	37.4%
	58–77	28	74	37.8%
	78–97	6	13	46.2%
	Total	162	386	42.0%
	Average age	44.5 years	43.0 years	
Without known family history of ATTRv	18–37	13	64	20.3%
	38–57	25	224	11.2%
	58–77	25	409	6.1%
	78–97	11	217	5.1%
	Total	74	914	8.1%
	Average age	57.8 years **	64.9 years	

Some individuals’ age was not available. * p-value < 0.05, ** p-value < 0.01.

The only study on the prevalence of ATTRv in Mallorca dates back to 2005, reporting a prevalence of 1 in 20,000 inhabitants^38^. A more recent study conducted in 2020, which included both symptomatic V30M ATTRv patients and asymptomatic carriers, estimated a prevalence of the V30M variant of approximately 1 in 4,800 inhabitants^17^. However, this estimate is likely conservative, as the cohort was limited to individuals treated at the island’s reference hospital for ATTRv, potentially excluding additional V30M carriers and thereby underrepresenting the true prevalence in the Mallorcan population. Furthermore, no prevalence data have been published for the other islands. To address this gap, the primary objective of this study was to establish an accurate registry and provide an updated estimate of the V30M prevalence across the Balearic Islands. To this end, we extended our analysis beyond Mallorca to include Menorca, Ibiza, and Formentera.

In addition to the 308 V30M carriers shown in Table 1 which were tested in our laboratory, there are an additional 124 documented historical cases of V30M ATTR individuals that were tested elsewhere over the past two decades and that are followed in the participating referral services, and one V30M carrier that was tested in a private entity (Genosalut). These 125 contribute to a total historical cohort of 433 individuals harboring the V30M variant, with 349 being currently alive. This updated analysis reveals a high prevalence of the V30M variant across the Balearic Islands (Table 3), which we estimate to be 1 in 3,400 inhabitants.

**Table 3:** Estimation of the prevalence of V30M ATTR in the Balearic Islands in 2023.

Island	V30M carriers	V30M carriers alive	Population 2023	Prevalence	
Mallorca	392	322	939,000	1 in 2,916	
Menorca	27	21	98,000	1 in 4,667	
Ibiza and Formentera	6	6	158,000	1 in 26,333	
Total	425^a^	349^a^	1,333,000	1 in 3,424	

^*a*^ Out of a total of 433 V30M carriers (symptomatic and asymptomatic), including 349 who are alive, information about their origin is missing in seven cases (one alive).

**Fig. 2:**
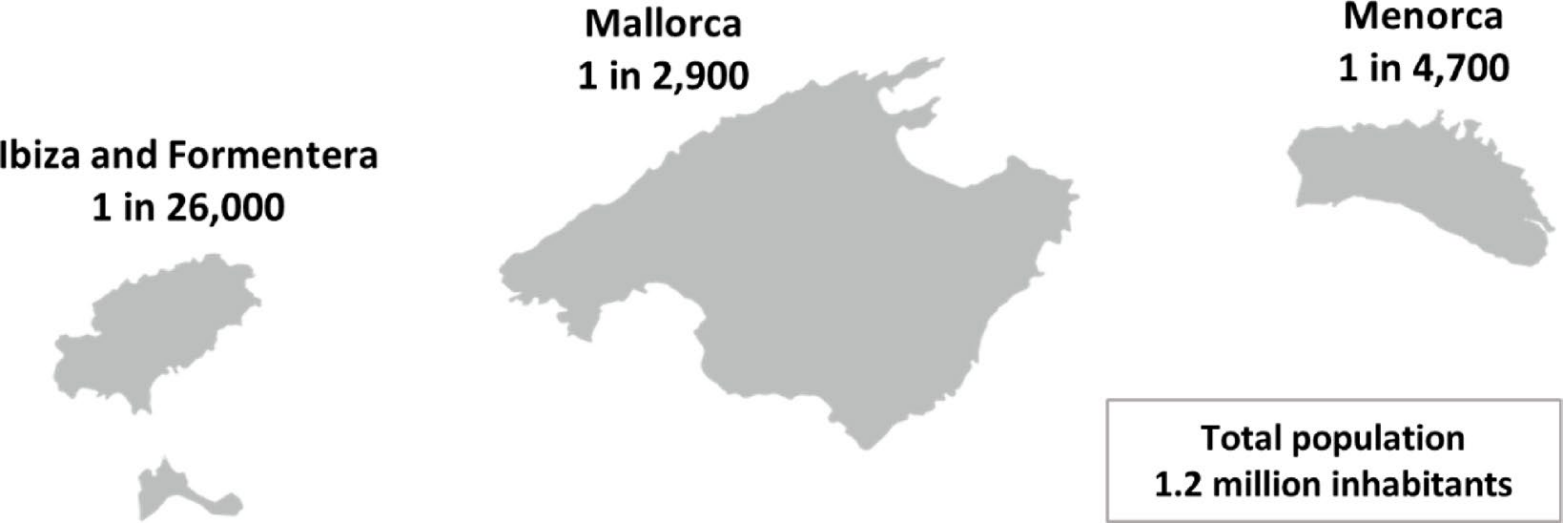
Geographical representation of the estimated prevalence of V30M ATTR in the Balearic Islands in 2023.

Breaking the data down by island (Fig. 2), Mallorca accounts for the largest number of affected individuals (*n* = 322), with an estimated prevalence of 1 in 2,900 inhabitants. Menorca, previously thought to have only isolated cases and a significantly lower prevalence than Mallorca^38^ now shows a substantial disease burden with a prevalence of approximately 1 in 4,700 inhabitants. Meanwhile, the V30M variant had not been documented in Ibiza or Formentera in prior studies. Our findings confirm its presence in these populations, although at a lower prevalence compared to Mallorca and Menorca of 1 in 26,000.

Data on other *TTR* variants of the 308 V30M carriers revealed that 93% of individuals carrying the V30M variant in exon 2 of the *TTR* gene also harbored the G6S variant, which is located in the same exon. This observation strongly suggests that both variants are carried on the same chromosome in a *cis* conformation. Genome sequencing further supported this hypothesis, as the two variants consistently appeared on the same sequencing reads (Supplementary Fig. 2).

**Fig. 3:**
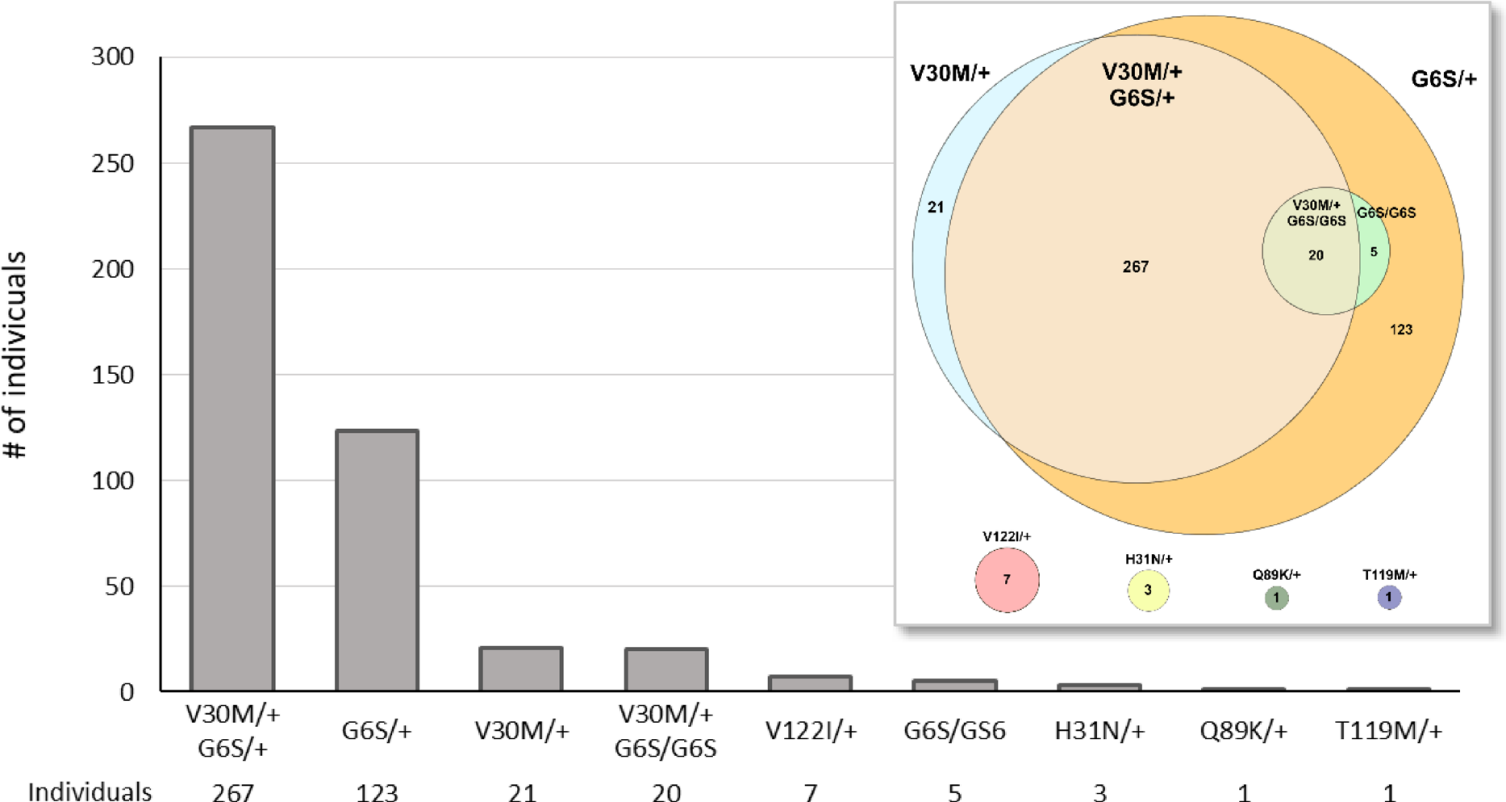
Graphical representation of the genotype classification of individuals with different ATTR variants identified in our cohort.

Interestingly, the *cis* configuration of G6S and V30M does not appear to have been previously described. The G6S variant, traditionally regarded as benign, has an allele frequency in the European population of 7.84% (92,438 out of 1,179,726 alleles) in the GnomAD 4.1 database and 7.85% (79 out of 1006 alleles) in the 1000 Genomes Project^39,40^. By comparison, the V30M variant is much rarer, with only 62 alleles reported in GnomAD and none in the 1000 Genomes Project^39,41^. Consistent with these data, our study identified a frequency of G6S (in the absence of V30M) in 10.9% (128/1170) of the tested population. Furthermore, only 21 V30M carriers from our cohort did not carry the G6S variant (*n* = 21; Fig. 3). Based on surname analysis, most of these non-G6S individuals likely have origins outside the Balearic Islands. All of this suggests that the majority of V30M carriers in our cohort are likely descendants of a single founder event in the Balearic Islands.

When we examined all other pathogenic *TTR* variants in our Balearic cohort, excluding both V30M and G6S, we identified 7 carriers of NP_000362.1:p.Val142Ile (V122I), 3 of NP_000362.1:p.His51Asn (H31N), and 1 each of Q89K and NP_000362.1:p.Thr139Met (T119M) (Fig. 3), corresponding to allele frequencies of 5.25·10⁻⁶, 2.25·10⁻⁶, 7.50·10⁻⁷ and 7.50·10⁻⁷, respectively. Among the individuals carrying non-Val30Met pathogenic or likely pathogenic *TTR* variants, 54.4% were male and 45.6% female. Three were identified as index cases with no known family history of amyloidosis, while seven were detected based on a positive family history; one individual’s familial status was unknown. The mean age at diagnosis for individuals without a family history was 76.3 years, compared to 46.0 years for those with a documented familial background.

## Discussion

Genetic testing for *TTR* gene variants is critical for the accurate diagnosis and management of ATTRv amyloidosis. This testing not only confirms the diagnosis but also enables personalized treatment, informed genetic counselling, improved prognostic assessment, and prevention of misdiagnosis. The identification of ATTR variants distinguishes the hereditary form (ATTRv amyloidosis) from other forms of amyloidosis, ensuring accurate classification of disease. Furthermore, targeted therapies, including ATTR stabilizers and *TTR* gene silencers, are based on the identification of pathogenic variants, allowing for treatment strategies tailored to the molecular underpinnings of the disease. Given the autosomal dominant inheritance of ATTRv amyloidosis, genetic testing facilitates family screening, enabling the identification of at-risk relatives. Early detection in these individuals allows for proactive surveillance and management, potentially mitigating the onset of severe symptoms. Additionally, knowledge of the specific ATTR variant provides prognostic insights, guiding clinical monitoring and management strategies to optimize patient care. Finally, testing helps differentiate ATTR amyloidosis from other conditions with similar clinical presentations, such as light-chain amyloidosis or immune polyneuropathies, thus avoiding misdiagnosis and inappropriate treatment^42^. In summary, *TTR* genetic testing is an indispensable tool in the comprehensive management of ATTRv amyloidosis, providing critical information for diagnosis, treatment, and familial risk assessment.

We present here a review of 23 years of symptomatic and presymptomatic genetic testing for ATTRv amyloidosis in the Balearic Islands, which has allowed a detailed characterization of the genetic, demographic and health features of ATTRv amyloidosis disease in this endemic focus. Our results emphasize the importance of family history in genetic testing outcomes. The diagnostic yield for V30M was markedly higher in individuals with a family history (42%) compared to those without (8.1%). The disparity in the timing of genetic testing further highlights the impact of family history on earlier identification. V30M carriers without a family history underwent genetic testing at a significantly older age compared to those with a family history (57.8 vs. 44.5 years, *p* = 6.71·10^− 6^). This delay in genetic testing among individuals without a family history reflects the limitations of relying solely on clinical presentation or generalized thresholds, such as a predicted age of disease onset (PADO) as proposed before^43^. Moreover, the co-occurrence of the V30M and G6S variants in 93% of V30M carriers suggests a potential founder effect, adding to the described genetic and phenotypic heterogeneity within ATTRv amyloidosis in general and V30M ATTR amyloidosis in particular. Age of onset and phenotypic traits have been described to be different among V30M individuals of different populations from different geographical locations^16,29^. Thus, all this complexity challenges the use of PADO as a universal metric for a specific variant, as different variants may have distinct origins and associated patterns of disease onset. Consequently, we highlight the need for early and flexible genetic testing strategies that do not rigidly adhere to age-based thresholds. Instead, individualized approaches that take into account family history and other contextual factors are essential to facilitate timely identification and proactive management of V30M carriers, ultimately improving patient outcomes.

The sex and age distribution among the individuals carrying the non‑V30M pathogenic variants (V122I, H31N and Q89K) in our cohort were comparable to those observed in the V30M group. In addition, the allele frequencies of these variants closely matched those reported in major population databases^39^. By contrast, T119M was observed at an allele frequency of 7.50·10⁻⁷, which is over three orders of magnitude lower than the 3.45·10⁻³ prevalence documented in reference datasets. The T119M variant is known as benign, and even acts as an ATTR tetramer‑stabilizing variant that protects against amyloid aggregation, reason why its carriers rarely develop symptomatic ATTR amyloidosis^44,45^. This protective effect likely explains why we identified only a single T119M carrier among individuals assessed for amyloidosis, despite its comparatively high population prevalence.

A recent genetic study of ATTRv amyloidosis in the United States reported that cardiology and neurology were the primary referral services for the ATTRv carriers, accounting for 93% of the tested individuals^46^. Similarly, in our findings, cardiology and neurology emerge as the main sources of referred patients. However, we also emphasize the significant roles of internal medicine and genetics. The testing referral reasons are primarily PST, polyneuropathy, and cardiac amyloidosis which align with the disease’s diverse clinical presentation impacting the nervous system, heart, gastrointestinal tract, and other organs.

Findings from a population-based cohort study of UK Biobank participants further underscore the importance of heightened clinical awareness in detecting *TTR* variants. The study identified that approximately 1 in 1000 participants carried likely pathogenic or pathogenic *TTR* variants, with a notably higher prevalence among individuals of African ancestry (4.3%), carrying mainly the p.Val122Ile variant, compared to those of European descent (0.02%). These likely pathogenic or pathogenic variants were significantly associated with adverse cardiovascular outcomes, including heart failure, conduction disease, and elevated mortality risk^47^. This data, combined with our own analysis of the Balearic Islands, where we found at least 0.033% of our population with a *TTR* variant, highlight the broader prevalence of pathogenic *TTR* variants and reinforce the need for systematic screening and early intervention strategies, particularly in high-risk populations. The findings support more rigorous clinical follow-up in endemic regions such as the Balearic Islands, where genetic diversity and founder effects may further complicate the clinical course of ATTR amyloidosis.

We observe a younger average age at genetic diagnosis for V30M carriers (57.8 years) compared to the overall testing age (64.9 years) among individuals without a known family history of ATTR amyloidosis. This difference may reflect a referral bias, as older individuals are more commonly tested due to the prevalence of neurological and cardiac conditions, while ATTRv remains a relatively rare cause of disease in such cases^48,49^.

We identified significant gender differences in test positivity for TTR variants, with women being more likely to test positive for the V30M variant than men. Despite more men being referred by cardiology and neurology, the gender bias is particularly evident in cardiology, where 25.0% of women tested positive for V30M compared to 7.8% of men, even though fewer women were referred. These findings align with a U.S. study reporting higher positivity for V30M among women tested, though overall case numbers were similar^46^. Possible explanations include referral patterns and healthcare disparities, as women (especially older women) may face biases that reduce their likelihood of being referred for genetic testing^50,51^. The higher prevalence of ATTRwt in men^18 ^along with their greater risk of cardiovascular disease, may contribute to more frequent testing among men^48^. In neurology, despite twice as many men being referred, the positivity rate was equal between genders. This disparity in referrals may be attributed to factors such as men being more prone to polyneuropathy^52^ or the potential influence of alcoholism^53^. However, other studies indicate no clear gender differences in polyneuropathy prevalence^54^. These results highlight the need to address gender bias in the detection of cardiac amyloidosis, particularly in our environment.

The Transthyretin Amyloidosis Outcomes Survey (THAOS) registry has provided global epidemiological data, including contributions from Spain, leading to a more comprehensive understanding of the prevalence and distribution of ATTRv. However, it also underscores the need for a national registry to better understand the epidemiology of this rare disease in Spain^18^. The findings presented here spanning two decades of symptomatic and presymptomatic ATTR amyloidosis testing provides nuanced understanding of the genetic landscape and epidemiology of ATTRv amyloidosis in the Balearic Islands. This has led to some unexpected findings highlighting Menorca as a significant endemic focus for the V30M variant along with Mallorca, a high prevalence of the disease, and a possible Balearic-specific founder effect.

With a prevalence of 1 in 2,900 in Mallorca and 1 in 4,700 in Menorca, the V30M variant occurs at much higher rates than previously reported^17,38,55^. However, our data reflects the variant prevalence (including both symptomatic and asymptomatic carriers), whereas the published prevalences of 1 in 700 in certain areas of Portugal and 1 in 1,000 in northern Sweden refer to ATTRv amyloidosis cases rather than asymptomatic carriers^11,56,57^. It is also important to note that our study relies on data from the public health system, which is a limitation, as diagnosed carriers identified through private institutions may not be included. As a result, our estimated prevalences could be underestimated. Although we did not directly assess clinical status, prior research in this population has demonstrated a penetrance exceeding 75% by age 90 ^58^, indicating that most V30M carriers will eventually develop ATTR amyloidosis. Taken together, these considerations place the Balearic Islands among the regions with the highest known V30M carrier frequencies, and given the high penetrance, likely correspond to one of the highest ATTRv amyloidosis burdens outside of the classic endemic foci. In contrast to the two major islands, the lower prevalence in Ibiza and Formentera (1 in 26,000) is consistent with the lack of previous documentation of V30M carriers, highlighting genetic differences between the islands within the archipelago, in line with a previously described different demographic history of Ibiza and Formentera with respect to Mallorca and Menorca^59^. Notably, the consistently high number of newly diagnosed individuals (Supplementary Fig. 1), coupled with advances in treatment efficacy, suggests that these prevalence rates may continue to increase. This trend likely reflects the growing awareness of ATTRv amyloidosis among clinicians in our region and a higher efficiency of genetic testing for the V30M variant in the Balearic Islands, due to a better selection of patients to be tested.

Our results challenge a previous hypothesis of a common genetic origin for the Balearic and Portuguese V30M carriers, as reported by Ohmori et al. in 2004^60^. We identified the co-occurrence of the V30M and G6S variants in 93% of V30M carriers, a pattern not reported in other V30M-endemic regions. The remaining 7% of carriers, without the G6S allele, had mostly non-Balearic surnames. Genome sequencing confirmed that these variants are in *cis* conformation on the same chromosome, reinforcing the likelihood of a shared ancestral origin of the V30M variant in the Balearic Islands. While this pattern is suggestive of a Balearic-specific founder effect, expanded and detailed haplotype analyses are still needed to confirm it. This may contradict the historical view and previous evidence suggesting a common genetic link between Mallorca and Portugal^60^. This distinct common origin, together with environmental factors, could be responsible for the differences observed in terms of disease manifestation, age of onset, and penetrance compared to other foci such as Portugal or Sweden^18,58^.

The high prevalence rates and the distinct genetic features highlighted in this study underscore the need for a region-specific approach to the management of ATTRv amyloidosis, including early genetic screening of at-risk individuals, increased awareness among clinician, and public health initiatives to ensure early diagnosis and intervention. Future research should focus on integrating clinical outcomes with genetic data to optimize treatment strategies and deepen our understanding of ATTRv amyloidosis in genetically diverse populations.

In conclusion, this two-decade retrospective analysis of genetic testing for pathogenic variants in the *TTR* gene highlights the significant prevalence of the V30M variant and ATTRv amyloidosis in the Balearic Islands, particularly in Mallorca and Menorca, identifying the region as potentially the third largest global focus for V30M cases after certain Swedish and Portuguese regions. Our findings underscore the unique genetic characteristics of ATTRv amyloidosis in this population, particularly the co-occurrence of the V30M variant with the benign G6S variant. This pattern strongly suggests a shared ancestral origin for nearly all V30M carriers of Balearic descent. Nevertheless, additional data and in-depth haplotype analyses are needed to definitively establish a distinct founder effect specific to the Balearic Islands, challenging previous hypotheses of a common origin with other V30M-endemic regions, such as Portugal.

## Supplementary Information

Below is the link to the electronic supplementary material.

Supplementary Material 1

Supplementary Material 2

Supplementary Material 3

## Data Availability

The datasets containing individual-level data that was generated in the current study are not publicly available, as patient privacy issues are involved in this study and there are ongoing follow-up studies, and the data are currently confidential but are available from the corresponding author on reasonable request. Whole genome sequencing data has been deposited in the Sequence Read Archive (SRA) database under the submission number SUB15174333 (BioProject: PRJNA1236698). Reviewer’s link to PRJNA1236698: https://dataview.ncbi.nlm.nih.gov/object/PRJNA1236698?reviewer=1lrb0nid20hnestiiju54c68ln.

## Abbreviations

E89K: NP_000362.1:p.Glu109Lys
G6S: NP_000362.1:p.Gly26Ser
H31N: NP_000362.1:p.His51Asn
T119M: NP_000362.1:p.Thr139Met
V122I: NP_000362.1:p.Val142Ile
V30M: NP_000362.1:p.Val50Met
PADO: Predicted age of disease onset
PST: Presymptomatic testing
ATTR: Transthyretin
THAOS: Transthyretin amyloidosis outcomes survey
TTR: Transthyretin “gene”
ATTRv: Variant transthyretin
ATTRwt: Wild type transthyretin
