# Supplementary material for: Genetic testing and evidence of a founder mutation in a hotspot for hereditary transthyretin amyloidosis
Source: Sci Rep. 2025 Aug 14;15:29773. doi: 10.1038/s41598-025-14707-4 (PMC12350801; doi:10.1038/s41598-025-14707-4)
Supplement: Supplementary file 1 — Supplementary Material 1 [file 41598_2025_14707_MOESM1_ESM.docx]

| ***Supplementary Table 1.*** *TTR variants identified in our cohort.* | | | | | | |
| --- | --- | --- | --- | --- | --- | --- |
| **Variant** | **HGVS (DNA)** | **HGVS (Protein)** | **rs ID** | **Consequence** | **Exon** | **# Carriers** |
| G6S | NM_000371.4:c.76G>A | NP_000362.1:p.Gly26Ser | rs1800458 | Missense (B/LB) | 2 | 415 |
| V30M | NM_000371.4:c.148G>A | NP_000362.1:p.Val50Met | rs28933979 | Missense (P) | 2 | 308 |
| H31N | NM_000371.4:c.151C>A | NP_000362.1:p.His51Asn | rs915983905 | Missense (VUS) | 2 | 3 |
| E89K | NM_000371.4:c.325G>A | NP_000362.1:p.Glu109Lys | rs121918082 | Missense (P/LP) | 3 | 1 |
| T119M | NM_000371.4:c.416C>T | NP_000362.1:p.Thr139Met | rs28933981 | Missense (B/LB) | 4 | 1 |
| V122I | NM_000371.4:c.424G>A | NP_000362.1:p.Val142Ile | rs76992529 | Missense (P/LP) | 4 | 7 |

B: Benign; LB: Likely Benign; VUS: Variant of Unknown Significance; LP: Likely Pathogenic; P: Pathogenic.
