# Supplementary material for: Genetic testing and evidence of a founder mutation in a hotspot for hereditary transthyretin amyloidosis
Source: Sci Rep. 2025 Aug 14;15:29773. doi: 10.1038/s41598-025-14707-4 (PMC12350801; doi:10.1038/s41598-025-14707-4)
Supplement: Supplementary file 2 — Supplementary Material 2 [file 41598_2025_14707_MOESM2_ESM.pptx]

## Slide 1
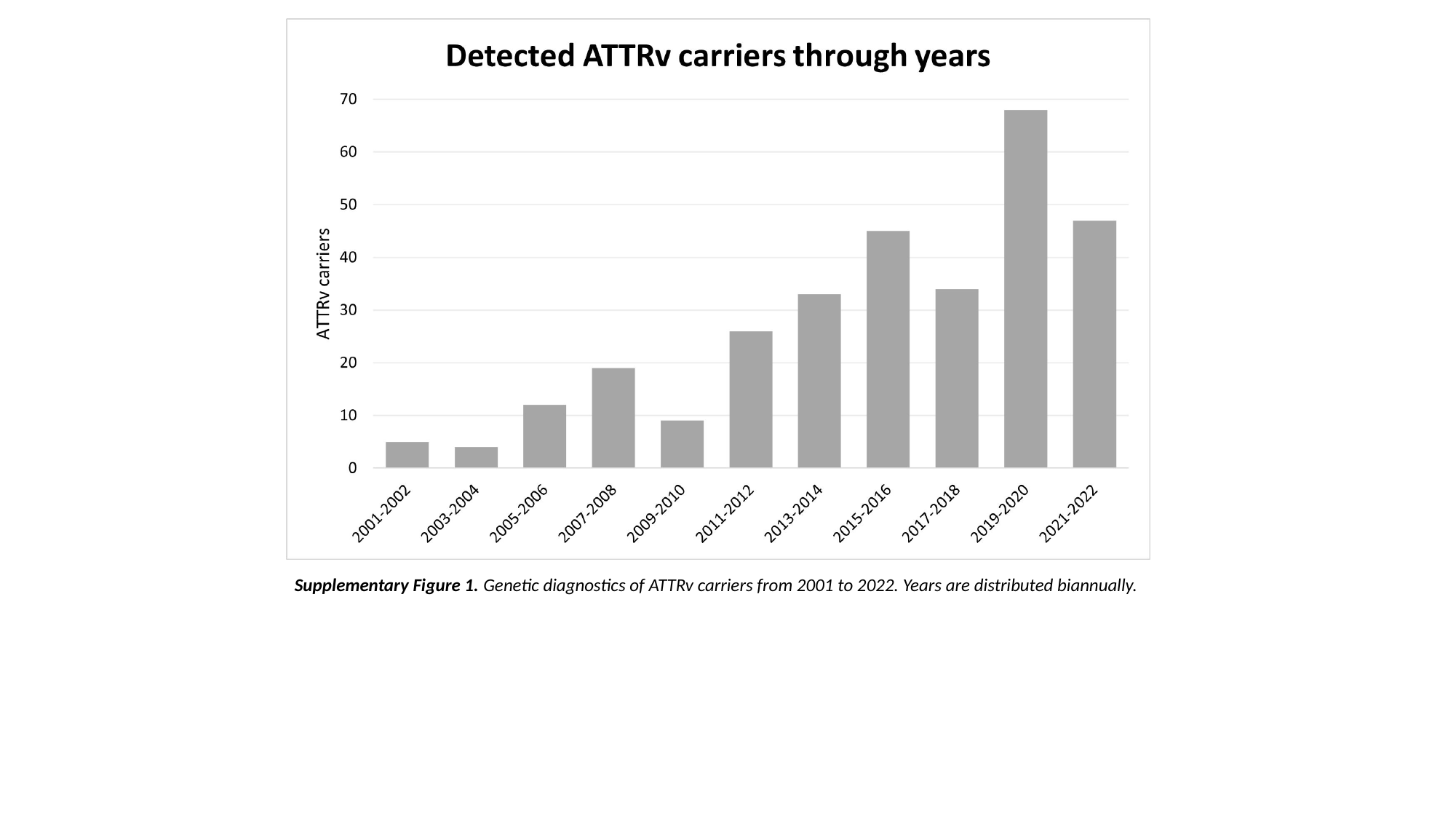

Supplementary Figure 1. Genetic diagnostics of ATTRv carriers from 2001 to 2022. Years are distributed biannually.
