# Supplementary material for: Genetic testing and evidence of a founder mutation in a hotspot for hereditary transthyretin amyloidosis
Source: Sci Rep. 2025 Aug 14;15:29773. doi: 10.1038/s41598-025-14707-4 (PMC12350801; doi:10.1038/s41598-025-14707-4)
Supplement: Supplementary file 3 — Supplementary Material 3 [file 41598_2025_14707_MOESM3_ESM.pptx]

## Slide 1
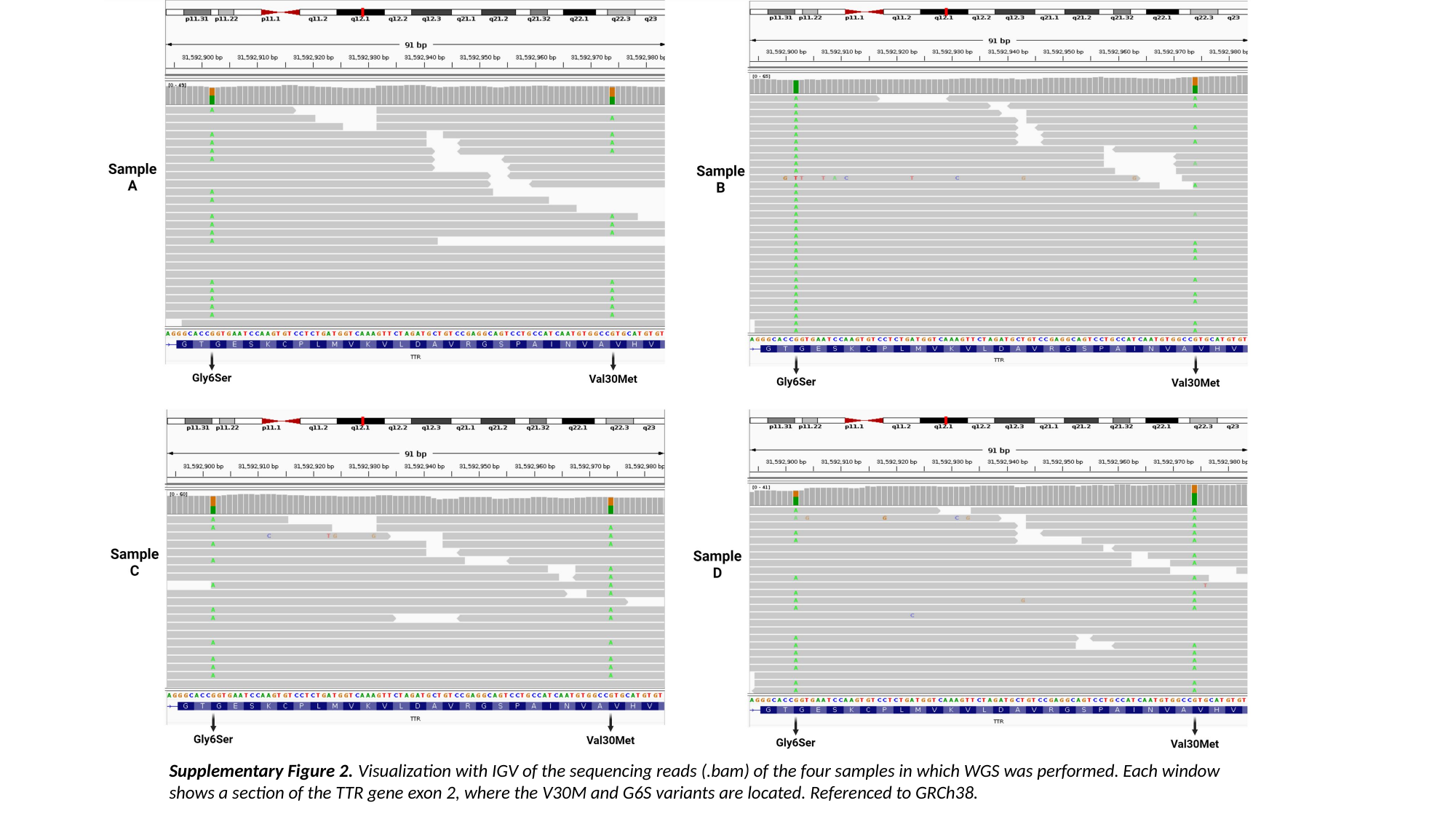

Supplementary Figure 2. Visualization with IGV of the sequencing reads (.bam) of the four samples in which WGS was performed. Each window shows a section of the TTR gene exon 2, where the V30M and G6S variants are located. Referenced to GRCh38.
